# Using an RNA Aptamer to Inhibit the Action of Effector Proteins of Plant Pathogens

**DOI:** 10.3390/ijms242316604

**Published:** 2023-11-22

**Authors:** Inna A. Abdeeva, Liliya G. Maloshenok, Gennady V. Pogorelko, Sergey A. Bruskin

**Affiliations:** 1 N.I. Vavilov Institute of General Genetics Russian Academy of Sciences, Gubkina Str. 3, 119333 Moscow, Russiabrouskin@vigg.ru (S.A.B.); 2 Department of Plant Pathology and Microbiology, Iowa State University, Ames, IA 50011, USA

**Keywords:** RNA aptamers, HopU1 protein, inhibition of protein activity, plant immunity, protein effectors, bacterial pathogens, transgenic plants

## Abstract

In previous work, we experimentally demonstrated the possibility of using RNA aptamers to inhibit endogenous protein expression and their function within plant cells In the current work, we show that our proposed method is suitable for inhibiting the functions of exogenous, foreign proteins delivered into the plant via various mechanisms, including pathogen proteins. Stringent experimentation produced robust RNA aptamers that are able to bind to the recombinant HopU1 effector protein of *P. syringae* bacteria. This research uses genetic engineering methods to constitutively express/transcribe HopU1 RNA aptamers in transgenic *A. thaliana*. Our findings support the hypothesis that HopU1 aptamers can actively interfere with the function of the HopU1 protein and thereby increase resistance to phytopathogens of the genus *P. syringae* pv. *tomato* DC 3000.

## 1. Introduction

Bacterial infection of plants causes enormous damage to agriculture. It is known that in order to infect the host plant, phytopathogenic bacteria are able to overcome both primary (PTI—Pathogen-Triggered Immunity) and induced (ETI—Effector-Triggered Immunity) immunity. To bypass plant PTI, the bacterial cells utilize effector proteins (Avr-proteins or avirulence proteins), which are delivered into plant host cells through the use of the bacterial type-III transport system (TTS). An example of this is observed in *P. syringae* infection where Hop proteins (Hrp outer proteins) suppress the PTI via several different mechanisms. The process of host immunity suppression is being actively studied, although the complex holistic scheme has not yet been established [1,2]. Moreover, it is readily known that when plant host cells are infected by effector proteins secreted by the bacterial type-III transport system (TTS), it triggers processes that allow the pathogen to manipulate the extracellular and intracellular environment of the host plant. This bacterial TTS mechanism promotes pathogen growth and development in the host plant [3,4].

In recent years, the functions of several proteins have been identified. In particular, the mechanism of the *P. syringae* HopU1 effector action was characterized. The HopU1 protein can suppress the innate immune responses that are triggered by either a type-III effector protein or a pathogen-associated molecular pattern [5]. HopU1 has been found to target certain mRNA-binding proteins, thereby suppressing the plant response to the infection. HopU1 is an active mono-ADP-ribosyltransferase that can modify arginine residues. It acts on at least two glycine-rich RNA-binding proteins (GR-RBPs), GRP7 and GRP8 [6]. GRP proteins play a positive role in plant immunity. There are studies that show that grp7 mutant plants show increased sensitivity to *P. syringae* pv. *tomato* DC3000 [7]. GRP7 has been shown to target RNA and influence messenger RNA oscillations in response to circadian rhythms at the post-transcriptional level [8]. Taking this into account, we assumed that the suppression of the HopU1 effector functions should lead to an increase in the resistance of plants to the action of a phytopathogen. As a tool for HopU1 effector functionality suppression, we propose using a novel tool such as RNA aptamers that bind specifically to this protein. Aptamers are nucleic acids, which are small fragments of DNA or RNA molecules that specifically bind to targets. Aptamers can be obtained using SELEX technology (Systematic Evolution of Ligands by Exponential Enrichment). Aptamers are synthesized to have high affinity and specificity to their target ligands. Aptamers can suppress the activity of proteins by binding to them in functionally critical sites [9,10]. Our earlier work demonstrated the efficient and effective use of RNA aptamers to inhibit the functions of plant endogenous proteins [11]. Therefore, this approach will likely be a robust method to inhibit exogenous foreign proteins that invade the host plant via various mechanisms, including phytopathogen effector proteins. To address the potential disadvantages of using RNA-like molecules, which are often unstable and susceptible to degradation, we propose creating endogenously expressed aptamers that will be transcribed by the plant cell transcription system during the entire life of the plant. This allows the plant host cells to maintain a quantifiable concentration of RNA aptamers. Secondly, our high-sensitivity aptamer selection method in transgenic plants will allow the localization of RNA aptamers to their complementary targets in the host plant cytoplasm, revealing any critical off-target effects.

## 2. Results and Discussion

The SELEX procedure included seven cycles. The double-stranded DNA pools selected during the fifth and the last seventh SELEX cycles were cloned into the pAL-TA vector (“Eurogen”, Moscow, Russia) and transformed into *E. coli* cells of the XL1blue strain. Plasmid DNA was isolated from 51 transformants containing aptamer sequences from the fifth round and 42 transformants from the seventh round. The size of the insertion was estimated via endonuclease restriction analysis. A total of 27 recombinant plasmids from the fifth cycle and 25 plasmids from the seventh cycle containing inserts of the correct size were sequenced. Paired analysis of the obtained sequences revealed six different groups of aptamers. All the sequences were split into two groups based on the identity degree: H1 and H2 (Table 1, Supplementary Table S1). In this regard, the most represented seed sequences from each group of aptamers were selected for synthesis and subsequent individual characterization. As a result, we have identified the nucleotide sequences of potential RNA aptamers.

The main indicator of the aptamer–target interaction specificity is the quantification of the complex dissociation constant (Kd), that is, the determination of the affinity of the aptamer for the target. The Kd measurement was carried out via the MST (Microscale Thermophoresis) method. This method is well suited for determining the dissociation constants of complexes and requires a small number of interacting molecules.

The MST method allows analysis in native biological conditions where various serums, lysates, and small sample volumes (um to nm) are required. This method allows the detection of significant changes in the solubility, charge, and size of molecules by measuring the molecular mobility in a temperature gradient. MST can be applied to detect a wide range of biomolecular interactions, from ions to large complexes (such as liposomes and ribosomes). The spectrum of measured dissociation constants is very broad, from pM to mM. The measurement itself is based on the direct movement of molecules along a temperature gradient, an effect called “thermophoresis”. The local temperature difference leads to a local change in the concentration of molecules, which is characterized by the Soret coefficient. Thermophoresis is monitored using a titer of fluorescent molecules in a permanent buffer. Since the buffer does not change, changes in thermophoresis can occur only due to alterations in the size, charge, or solubility of the fluorescent molecules. The thermophoretic movement of a fluorescent molecule is measured by monitoring the distribution inside the capillary with a Nano Temperature Monolith device. A microscopic temperature gradient is created using an IR laser that focuses on the capillary and is strongly absorbed by water. Heating occurs at the place of laser exposure and the molecules begin to move from the heated area to a cooler one, and their localization varies depending on the speed of movement, which depends on their size.

**Figure 1:**
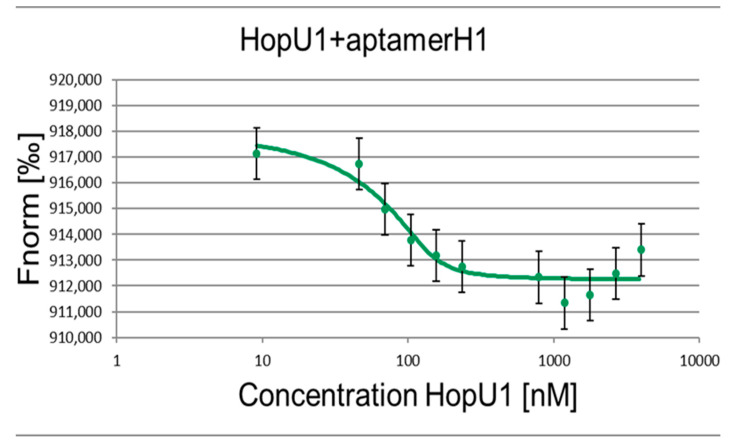
Binding of the H1 RNA aptamer to the HopU1 protein. The concentration of the fluorescently labeled H1 RNA aptamer 50 nM, the concentration of the HopU1 protein 10–10,000 nM.

**Figure 2:**
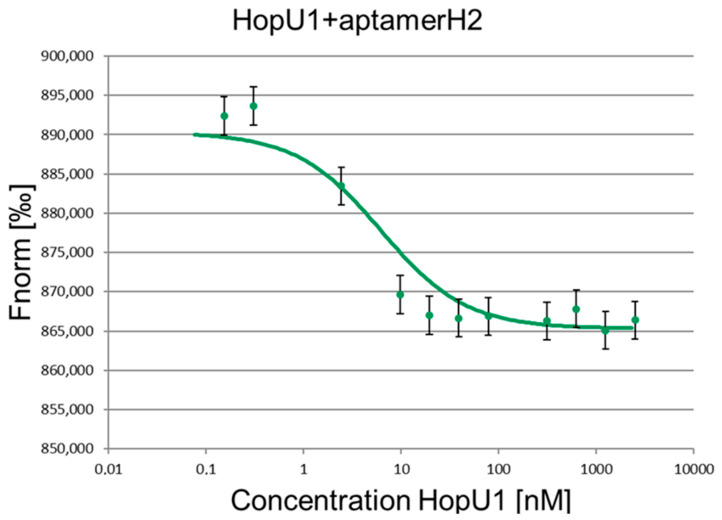
Binding of aptamer H2 to the HopU1 protein. The concentration of the fluorescently labeled RNA aptamer H2 50 nM, the concentration of the HopU1 protein 0.2–10,000 nM.

To carry out the Kd measurements for the selected H1 and H2 RNA aptamers, RNA oligonucleotides were labeled with a fluorescent label FAM (carboxyfluorescein) attached to the 5′ end. The concentration of fluorescently labeled aptamers was constant and equal to 50 nM, while the concentration of the HopU1 protein varied. Several dilutions of HopU1 in buffer A (25 mM Tris HCl; 10 mM NaCl; 25 mM KCl; 25 mM MgCl_2_; pH 8.0) were used for analysis in the range from 10 uM to 100 pM. Each dilution contained 50 nM RNA aptamer. The prepared samples were placed in the capillaries and analyzed using a Nano Temper Monolith machine to obtain the Kd values. Table 2 and Figure 1 and Figure 2 present the measured dissociation constants of aptamers H1 and H2. Bovine serum albumin (BSA) was used as a negative control binding agent to confirm the specificity of the H1 and H2 aptamers’ interaction with the HopU1 protein (Supplementary Figure S1). As an additional control, we accessed the HopU1 protein’s binding possibility to an unrelated GFP–aptamer to eliminate the possibility of HopU1 non-specific binding to any aptamer sequences (Supplementary Figure S2).

The measured Kd for H1 and H2 aptamers are consistent with the literature data for other RNA aptamers. Usually, the dissociation constants of protein–RNA aptamer complexes lie in the range of 10^−9^–10^−11^ M, which is an indicator of the high affinity of the aptamer for the target molecule. For example, the Kd of the aptamer–thrombin complex is 0.1 nM (aptamer N = 29) and 100 nM (aptamer N = 15) [12], while the Kd of the antigen–antibody complex for RgrS is 0.1–10 nM for PrPSc 1 uM [13].

**Figure 3:**
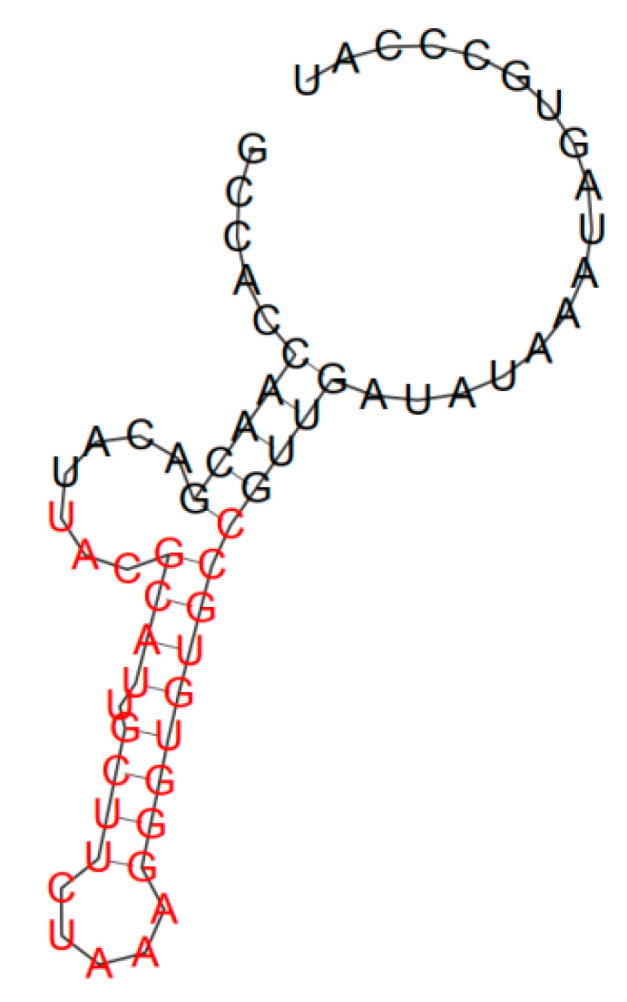
Secondary structure of the H2 aptamer.

**Figure 4:**
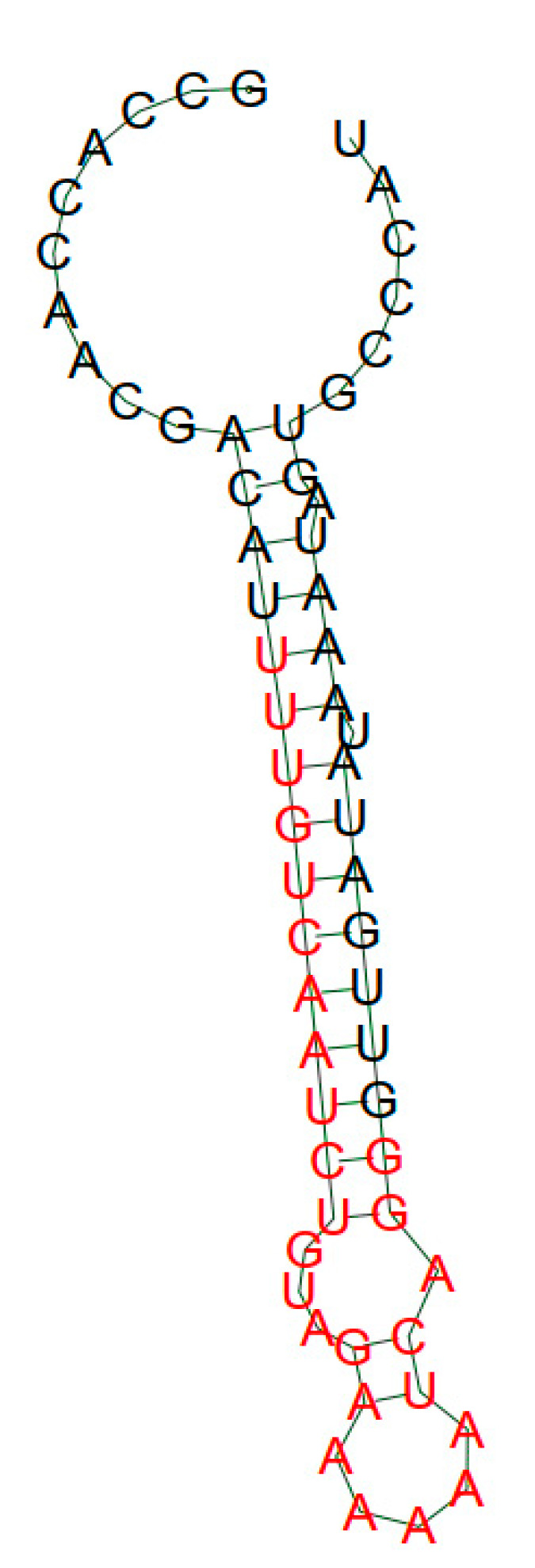
Secondary structure of the H1 aptamer.

A characteristic feature of aptamers is the ability to form a pronounced secondary structure. In many cases, it has been demonstrated that critical aptamer–target interactions occur at sites where aptamers form a stable secondary structure necessary for maintaining the correct interposition of recognition elements [14,15]. Many free-form aptamer sites are unstructured (for example, loops and hairpins) and acquire a stable conformation only after binding to the target [16,17]. Secondary structures of the selected HopU1 protein aptamers were predicted using Mfold software (http://rna.tbi.univie.ac.at (accessed on 9 October 2023)). The structures with the minimal free energy of aptamers are shown in Figure 3 and Figure 4. Aptamer H2 has a secondary structure with a loop cycle and an internal convexity (Figure 3). The bulge is surrounded by two regions of duplex RNA of seven and three base pairs. Of the 61 bases, 26 are in base pairs and 35 are single-stranded. The aptamer has a high predicted stacking stability, with a minimum free energy of ∆G = −11.55 kcal/mol at 22 °C. As for the secondary structure of the aptamer H1 (Figure 4), with the minimum free energy of ∆G = −13.16 kcal/mol at 22 °C, it has an internal bubble and a hairpin with five “A” loops. The H1 predicted folded structure consists in total of 34 complementary bound nucleotides and 27 unbound free bases.

Expression vector constructs for the constitutive transcription of the H1 and H2 RNA aptamers in *A. thaliana* plants were created on the basis of the pCXSN plasmid [18]. Obtained via the floral dip method, *A. thaliana* transgenic plants were subjected to selection on the hygromycin-containing plant media followed by genomic DNA testing for transgene presence by PCR. More than five independent plant lines were obtained for each vector construct carrying either the H1 or H2 aptamer sequence. The resulting transgenic plants had a phenotype similar to the control wild-type Arabidopsis plants.

**Figure 5:**
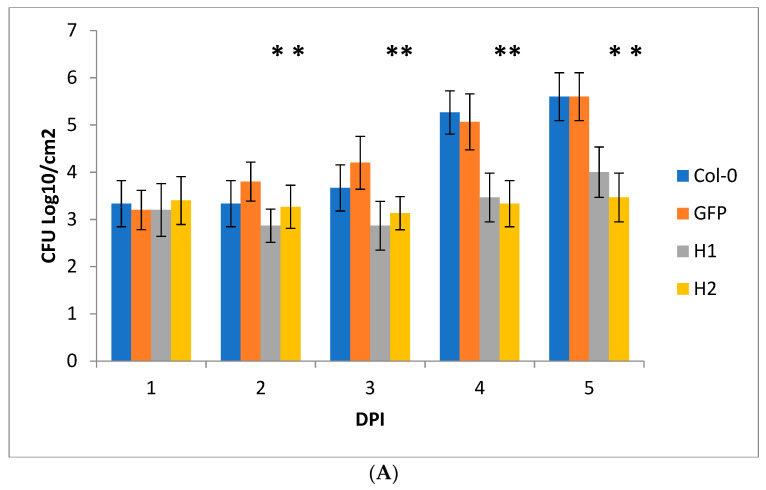

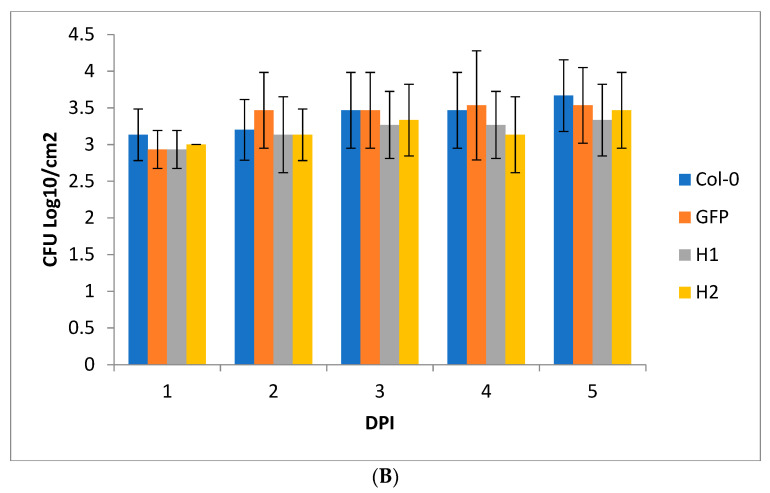
(**A**) The growth of the population of bacterial cells *P. syringae* pv. *tomato* DC3000 in the leaves of transgenic plants *A. thaliana* (lines H1 and H2) compared with the control plants ecotype *Col*-0 and GFP–aptamer-expressing plants at certain time intervals after inoculation. Infiltration was carried out via suspension culture with a concentration of 10^5^ cfu/mL. Three technical repeats per each transgenic line. Five independent transgenic lines per each H1 or H2 aptamer. The diagram shows the mean and standard deviations. Asterisks indicate the Student’s criterion test, *n* = 3; *p* < 0.01. (**B**) The growth of the population of bacterial cells *P. syringae* ∆*hopU1mutant* in the leaves of the transgenic plants *A. thaliana* (lines H1 and H2) compared with the control plants ecotype *Col*-0 and GFP–aptamer-expressing plants at certain time intervals after inoculation. Infiltration was carried out via suspension culture with a concentration of 10^5^ cfu/mL. Three technical repeats per each transgenic line. Five independent transgenic lines per each H1 or H2 aptamer. The diagram shows the mean and standard deviations. Asterisks indicate the Student’s criterion test, *n* = 3; *p* < 0.01.

**Figure 6:**
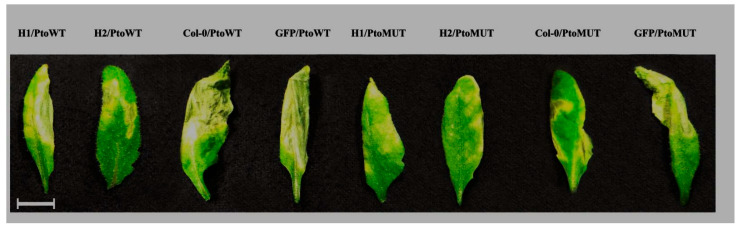
Disease symptoms of transgenic lines expressing the H1 and H2 aptamers and in Col-0 and GFP control plants at 5 days post-inoculation with *P. syringae* pv. *tomato* DC3000 wild-type (PtoWT) and *P. syringae* ∆*hop*U1 mutant strain (PtoMUT). Bar = 1 cm.

To determine the effect of the transcription of the H1 and H2 aptamers on the triggering of tolerance to *Pseudomonas* infection, the leaves of selected transgenic plants were infected with *P. syringae* pv. *tomato* (Pss) bacteria, followed by an assessment of the pathogen population rate (cfu/cm^2^ of the leaf surface at various time intervals after inoculation). To confirm that the difference in the infection response is caused by effector-targeting aptamer expression, the control plants of *Arabidopsis* expressing GFP-specific aptamer were used. GFP–aptamer *Arabidopsis* transgenic plants were created with the use of the pCXSN-Apt-GFP vector and stable homozygous transformants [11]. As an additional control, the *P. syringae* DC3000 ∆*hop*U1 mutant was used in the infection assays. The results shown in Figure 5A reveal that during the first 24 h, there were no significant differences in the development of *P. syringae* wild-type infection between the transgenic lines and *Col*-0 or GFP–aptamer plants; however, at later stages, the growth of the pathogen population in the control plants increased significantly. Such observations could be caused by the fact that at the first stages of the development of infection, an increase in the mass of bacterial cells occurs due to the colonization of the apoplast, and only when a certain population density is reached does the invasion into the host plant cell begin. The infection with the *P. syringae* ∆*hopU1* mutant strain did not reveal a significant difference in the infection rate between the H1, H2 and control plants (Figure 5B). The diagram in Figure 5 and the observed development of the disease symptoms in Figure 6 show *A. thaliana* transgenic plants transcribing H1 and H2 aptamers, with statistically significant increased resistance to the action of the wild-type strain phytobacterial pathogen *P. syringae* when compared to the control plants. For the H2 transgenic lines, when compared to the Col-0 and GFP–aptamer plants, there is more than a 100-fold difference in the growth of *P. syringae* bacteria. In the case of the H1 line plants, the difference in the growth of *P. syringae* bacteria compared to the control plants is less than 100-fold during the entire monitoring period of 5 days post-inoculation (Figure 5A). These data suggest that aptamer H2 suppresses the functional activity of the HopU1 protein more strongly than H1, and in case of infection with the *P. syringae* ∆*hopU1* mutant strain where the target for H1 and H2 aptamers is missing, the difference between the aptamer-expressing plants and control plants is neglected.

**Figure 7:**
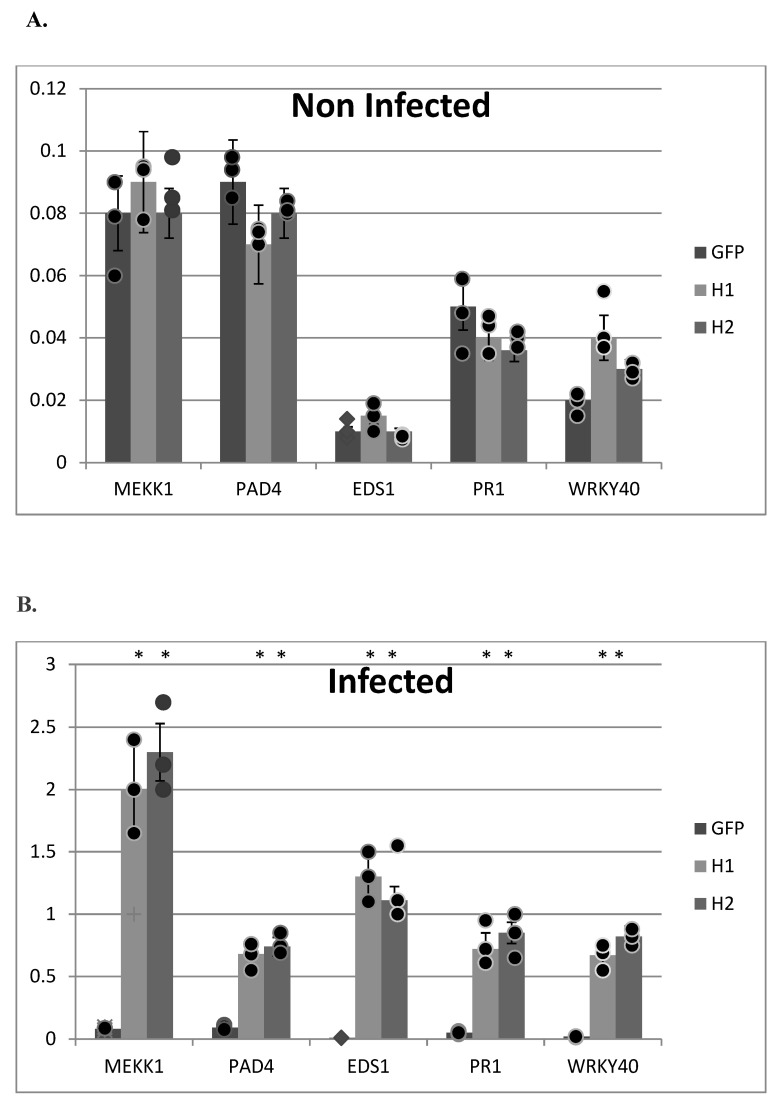
Real-time RT–PCR analysis of selected pathogen-related genes in H1, H2, GFP transgenic lines non-infected (**A**) and infected (**B**) with *P. syringae* pv. *tomato* DC3000 at 5DPI. Gene expression levels in transgenic plants normalized to the expression of the same gene detected in wild-type Col-0 plants (for which gene expression was set to 1). Actin2 gene was used as reference gene. The comparative threshold cycle method was used for determining differences between transcript copy numbers in wild-type and transgenic plants. Data represent average obtained for three independent transgenic plants. Asterisks indicate significant differences between transgenic plants and wild-type plants (Student’s *t* test, *p* < 0.05; *n* = 3).

To confirm the H1 and H2 aptamers’ potential to restrict the suppression of plant immunity by *Pseudomonas*, we assessed the transcript levels of the general defense marker gene *MEKK1* (*MAPK/ERK kinase kinase member A1*), the marker genes of the salicylic acid signaling pathway *PAD4* (*Phytoalexin Deficient 4*), *EDS1* (*Enhanced Disease Susceptibility 1*), *WRKY40* (*WRKY DNA-binding protein 40*) and *PR1* (*Pathogenesis-Related 1*), as well as the jasmonate-responsive plant defensin gene *PDF1.2* in the leaf tissues of non-infected and infected plants. We observed statistically significant up-regulation of these marker genes in the H1 and H2 infected plants (Figure 7B). The control GFP–aptamer-expressing line revealed no significant difference compared to the wild-type Col-0 plants. Non-infected plants were not showing altered transcript levels, suggesting that the H1 and H2 aptamers are not triggering plant defense mechanisms by themselves.

## 3. Material and Methods

### 3.1. HopU1 Preparations

**Figure 8:**
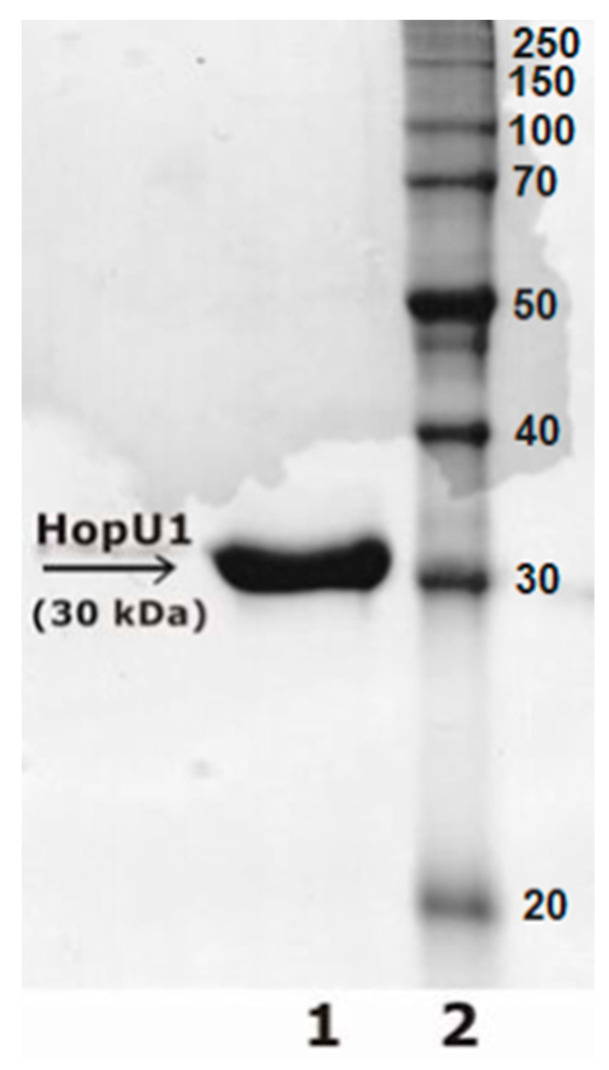
12% PAGE of HopU1 protein. 1-purified HopU1 protein (30 kDa); 2–5 μg Prestained Protein Ladder (Thermo Fisher, Cat# 26630, Waltham, MA, USA).

The sequence of the hopU1 gene encoding the *P. syringae* HopU1 effector protein was amplified from the genomic DNA via PCR using a pair of primers: “HopU1-ForwNdeI+” 5′-CATATGAATATAAATCGACAACTGCCTG-3′ and “HopU1-RevXhoI-” 5′-CTCGAGAATCTGACTTAATACAAATAAATGC-3′. A PCR fragment pre-treated with NdeI–XhoI restriction enzymes was cloned into the *E. coli* expression plasmid pET22b (+) pre-treated with the same endonucleases (Novagen, Madison, WI, USA). The correctness of the expression vector assembly was confirmed via sequencing. Protein production was carried out in the *Rosetta* 2 (DE3) (Novagen) *E. coli* strain for 4 h after the induction of 1 mM IPTG (isopropyl-β-D-1-thiogalactopyranoside) at a temperature of 37 °C. The recombinant protein HopU1 was purified using Ni-NTA agarose (Qiagen, Singapore). The cell culture containing the accumulated recombinant protein was lysed in 100 mM NaH_2_PO_4_, 300 mM NaCl, 8 M urea buffer at pH = 8.0 and applied to a Ni-NTA resin for 16 h at 10 °C. Then, the Ni-NTA column was washed several times with 100 mm NaH_2_PO_4_, 300 mM NaCl and 10 mM imidazole at pH =8.0 until the protein concentration in the wash fractions reached OD600 = 0.05 or less. The recombinant protein was washed from a column of 100 mm NaH_2_PO_4_, 300 mM NaCl and 300 mM imidazole buffer at pH = 8.0. The protein concentration was measured using the Qubit Protein Assay Kit. Protein analysis was performed in 12% PAGE (polyacrylamide gel electrophoresis) stained with Coomassie blue (0.1% (*w*/*v*) Coomassie blue R350, 20% (*v*/*v*) methanol, and 10% (*v*/*v*) acetic acid) (Figure 8). The protein band at 30 kDa mass was extracted from the gel and subjected to mass spectrometry. The resulting protein was transferred to binding buffer A (Tris HCl 25 mM; NaCl 10 mM; KCl 25 mM; MgCl_2_ 25 mM; pH 8.0) until necessary for further use in the SELEX procedure.

### 3.2. Mass Spectrometry (LC–MS)

**Figure 9:**
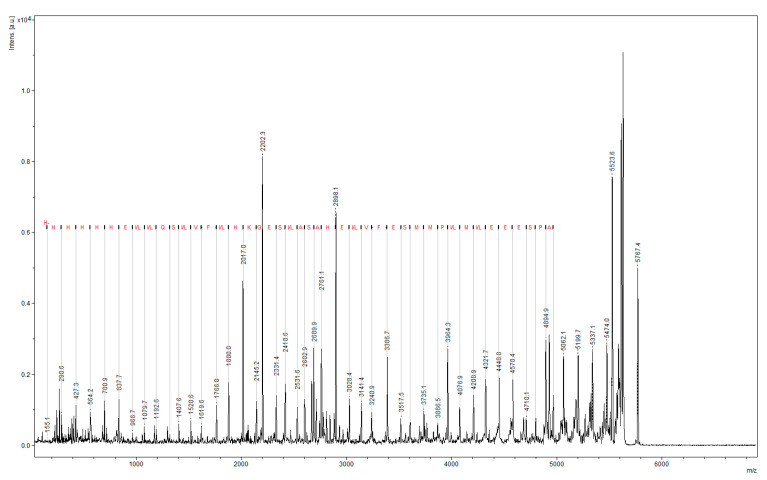
Results of mass spectrometric analysis of the recombinant protein HopU1 sequence.

Gel slices with the HopU1 protein were minced, washed with deionized water, destained with 100 mM ammonium bicarbonate (ABC), acetonitrile, and dried with acetonitrile before the reduction step. Reduction and alkylation were performed, respectively, with 5 mM Tris(2-carboxyethyl) phosphine hydrochloride (TCEP) in 100 mM ABC pH 8.6 for 30 min at 37 °C. Incubation with 50 nM iodoacetamide (IDA) in 100 mM ABC for 30 min in dark at 37 °C. Next, 0.5 µg/µL of sequencing-grade trypsin (Promega, Madison, WI, USA) was added to the gel spot. The samples were incubated at 37 °C in a shaker for 19 h, and then the peptides were extracted three times via exchange with 20 µL aliquots of 5% formic acid and 50% acetonitrile at room temperature for 20 min each. The sample was used for LC–MS analysis. The amino acid composition of the resulting protein was determined via mass spectrometry (Figure 9).

LC–MS analysis confirmed that the amino acid sequence of the 30 kDa expressed protein matches the *P. syringae* HopU1 protein.

### 3.3. SELEX

The SELEX procedure was performed according to the established protocol [19] with modifications.

Transcription templates were synthesized via PCR using synthetic oligonucleotides: “Library” 5′-TTACAGCAACCACCGGGGATCCATGGGCACTATTTATATCAAC (N)25 AATGTCGTTGGTGGCCC-3′, where N- is a random nucleotide. The PCR primer sets used were: N25 template “Forward” 5′-CGCGAATTCTAATACGACTCACTATAGGGGCCACCAACGACATT-3′, where T7 promoter sequence is underlined, “Reverse” 5′-CCCGACACCGCGGGATCCATGGGCACTATTTATATCAA-3′.

The N25 RNA pools were prepared via in vitro transcription with T7 RNA polymerase (Fermentas, Vilnius, Lithuania). RNAs were refolded via heat denaturing (at 85 °C) and slow cooling (to room temperature) in buffer A (Tris HCl 25 mM; NaCl 10 mM; KCl 25 mM; MgCl_2_ 25 mM; pH 8.0). Selections were performed in buffer A containing 100 units RNase inhibitor (Fermentas, Vilnius, Lithuania) and 10 mM ATP.

In the selection, HopU1 (10 µg) was mixed with 20 µL of Ni-NTA agarose (Qiagen; pre-equilibrated with buffer A) at +4 °C for 2 h. After washing out the unbound protein, each round RNA pool (50 µL) was added and incubated for 4 h at +4 °C temperature. The RNA–protein complexes were isolated via centrifugation and washing (buffer A +10 mM imidazole) and eluted with buffer A containing 300 mM imidazole. RNAs were extracted with phenol/chloroform treatment and precipitated with ethanol. Then, cDNAs were synthesized with SuperScript III Reverse Transcriptase (Invitrogen, Cat# 12574026, Waltham, MA, USA) and N25 forward primer 5′-CGCGAATTCTAATACGACTCACTATAGGGGCCACCAACGACATT-3′, amplified via PCR using the N25 primer set and followed by T7 transcription. The RNA was used for the next round of selection. An initial molar ratio of 3 µM protein to 6 µM RNA (1:2) was raised to 0.3 µM protein to 4.5 µM RNA (1:15) in the final round.

Before the first SELEX round, a control step was introduced (using an empty Ni-NTA agarose without any protein), which discarded aptamers that could have been non-specifically bound to the inert surface. Selection-amplification was repeated 7 times. A total of 7 selection cycles were performed. cDNAs obtained after the 7th round were cloned into the pAL-TA plasmid (Eurogen, Moscow, Russia) and sequenced.

The selected H1 and H2 RNA sequences were transferred into the plant expression T-vector pCXSN [18] and then pretreated with restriction endonuclease XcmI. The recombinant plant vectors pCXSN-H1 and pCXSN-H2 were confirmed via sequencing and were used to transform the agrobacterial cells of the GV3101 strain.

### 3.4. Agrobacterial Transformation of A. Thaliana Plants

The agrobacterial transformation of *A. thaliana* Columbia-0 ecotype plants with the vector constructs pCXSN-H1 and pCXSN-H2 was carried out via the floral dip method. This method refers to vacuum infiltration methods [20]. To carry out the transformation, the plants were grown up to the stage of formation of a large number of dustless green buds (approximately 3–4 weeks). The transformation was preceded by the pruning of all the formed pods as well as open flowers. The shoots of plants with immature buds prepared in this way were completely immersed in 1 L of a solution containing 5% sucrose, 0.05% MES, 200 µL Silwet L-77, pH = 5.7, and *A. tumefaciens* cells culture at OD600 = 0.5. The obtained seeds from the transformed plants were germinated in the presence of antibiotic hygromycin (30 mg/L), and after 14 days of selection, the resistant plants were transferred to regular soil. To confirm the transgenicity of the plants, the genomic DNA was isolated from the leaves using CTAB1 (Cetyltrimethylammonium Bromide) reagent and subjected to PCR verification using primers specific for HopU1 coding sequences (“35SP_seq” 5′-GCAAGTGGATTGATGTGATATC-3′, “H2-forw” 5′-TACGCATTGCTTCTAAAGGGTGTGCC-3′, “H1-forw” 5′-TTTGTCAATCTGTAGAAAAAATCAGG-3′).

### 3.5. P. syringae hopU1 Mutant Creation

A *P. syringae* ∆*hopU1* mutant was created by amplifying a 2 kb upstream and downstream region of hopU1 using PCR with the primer sets Up-F (5′-ATGAGGATCCGGATGGGCATGCTCGAAG-3′) + UpR (5′-ATGAAAGCTTAGGCAGTTGTCGATTTA T-3′) and DoF (5′-ATATCTAGAGTCCATGAAGGAGGCCGTACG-3′) + DoR (5′-ATGAGAGCTCGCCTGTCACGACGCCACT -3′) containing BamH1, HindIII, XbaI and SacI restriction sites, respectively. The *hopU1* upstream DNA fragment was ligated into pHP45Ω [21] using the BamHI and HindIII sites. The DNA fragment downstream of *hopU1* was ligated into the pHP45Ω derivative containing the upstream fragment using the XbaI and *Sac*I restriction enzymes such that the *hopU1* flanking regions were on either side of an Ω fragment in the same orientation. This cassette was subcloned into the broad-host-range vector pRK415 [22] using BamHI and SacI restriction endonucleases. The resulting construct was transformed into DC3000 via electroporation and homologous recombination was selected with the use of the antibiotic marker linked to the mutation and loss of the plasmid marker. The obtained mutant was confirmed via PCR using flanking region-specific primers.

### 3.6. Analysis of the Transgenic Arabidopsis Plants’ Resistance to the Action of Phytopathogens

To test the resistance of *A. thaliana* transgenic plants to infection with *P. syringae* pv. *tomato str*. DC3000 phytopathogens, a technique developed and optimized by us earlier was used. Plants were grown under optimal conditions for 5 weeks. Before treatment, the plants were kept in the dark for 16 h. Infiltration of the *A. thaliana* plants by bacterial cells of the phytopathogen *P. syringae* was carried out on the underside of the leaf plate at a concentration of 10^5^ cfu/mL in a buffer solution (10 mM MES, 10 mM MgCl_2_, pH 5.8) using a needleless syringe according to the common protocol described by Swanson [23]. The rate of bacterial infection was determined by counting the CFU (colony forming units) per unit of leaf surface at certain time intervals (from 1 h to 5 days). To measure the cfu/cm^2^, a fragment of a leaf disk with an area of 50 mm^2^ at the infiltration site was ground in 1 ml of 10 mM MgCl_2_ (a buffer solution that does not affect the viability of bacterial cells), and then a series of dilutions of the resulting suspension was obtained from 10^−1^ to 10^−8^. The aliquot of each dilution was sown on an LB medium with a selective agent—rifampicin 50 mg/L—followed by counting the grown colonies.

### 3.7. Quantitative Real-Time PCR

The total RNA was extracted from at least 3 separate biological replicates for each experiment using the PerfectPure RNA Fibrous Tissue Extraction Kit (5 Prime) (Thermo Fisher Scientific, Waltham, MA, USA) according to the manufacturer’s instructions. The PCR reactions were run in an I-Cycler (Bio-Rad) using the following program: 50 °C for 10 min, 95 °C for 5 min, and 40 cycles of 95 °C for 30 s and 56 °C for 30 s using gene specific primers (Supplementary Table S2). Following the PCR amplification, the reactions were subjected to a temperature ramp to create the dissociation curve, as determined by the changes in the fluorescence measurements as a function of the temperature, by which the non-specific products can be detected. The expression levels were calculated using the 2^−ΔΔCT^ method.

## 4. Conclusions

The use of endogenously expressed RNA aptamers to increase the resistance of plants to bacterial infection by inhibiting the functions of individual effector proteins is a promising and universal way to protect plants from a variety of pathogens, including but not limited to bacteria. RNA aptamers can also be effectively targeted toward insects, fungi, and nematode pests. With the use of modern genetic engineering and bioinformatics technologies, transgenic plants with the constitutive transcription of specific and highly efficient aptamers–inhibitors of effector proteins can be created and used in agriculture. Our results indicate that the use of RNA aptamers is a promising and effective method for targeting proteins within a plant cell in vivo. Support for this is shown in our previous work, where the suppression of the green fluorescent protein (GFP) in transgenic plants constitutively expressing the GFP gene was achieved via co-expression of the GFP-targeting RNA aptamer. In the current work, we have proved the applicability of the RNA–aptamer-based method as an efficient pest control tool that can be presumably used in a variety of plant species to protect them from a broad spectrum of plant pathogens.

## Figures and Tables

**Table 1:** Nucleotide sequences of aptamers used for synthesis (the variable part of aptamer N25 is highlighted in bold italics and contains “U” instead of “T” in the RNA version of the aptamers).

Aptamer ID	Sequence
H1	5′-GCCACCAACGACAT*TTTGTCAATCTGTAGAAAAAATCAGG*GTTGATATAAATAGTGCCCAT-3′
H2	5′-GCCACCAACGACAT*TACGCATTGCTTCTAAAGGGTGTGCC*GTTGATATAAATAGTGCCCAT-3′

**Table 2:** Dissociation constants (Kd) of RNA aptamer complexes with HopU1 protein.

Aptamer	H1	H2
Kd, nM	6.71 ± 0.69	6.41 ± 0.48

## Data Availability

Data are contained within the article and Supplementary Materials.

## Supplementary Materials

The supporting information can be downloaded at: https://www.mdpi.com/article/10.3390/ijms242316604/s1.
