# Supplementary material for: Using an RNA Aptamer to Inhibit the Action of Effector Proteins of Plant Pathogens
Source: Int J Mol Sci. 2023 Nov 22;24(23):16604. doi: 10.3390/ijms242316604 (PMC10705891; doi:10.3390/ijms242316604)

Table S1. Clustered sequencing results of aptamers obtained in 5th and 7th cycles of SELEX. Red "apt" corresponds to the sequences from 5th cycle. Black "hop" corresponds to the sequences from 7th cycle.

Number of sequences	Aptamer ID	Sequence
		10 20 30 40 50 60 70
		....|....|....|....|....|....|....|....|....|....|....|....|....|....|
	hop 1	hop 1 GGGATCCATGGGCACTATTTATATCAACCCTGATTTTTTCTACAGATTGACAAAATGTCGTTGGTGGCCC
	hop 9	hop 9 GGGATCCATGGGCACTATTTATATCAACCCTGATTTTTTCTACAGATTGACAAAATGTCGTTGGTGGCCC
	hop 10	hop 10GGGATCCATGGGCACTATTTATATCAACCCTGATTTTTTCTACAGATTGACAAAATGTCGTTGGTGGCCC
	hop 19	hop 19 GGGATCCATGGGCACTATTTATATCAACCCTGATTTTTTCTACAGATTGACAAAATGTCGTTGGTGGCCC
	hop 20	hop 20 GGGATCCATGGGCACTATTTATATCAACCCTGATTTTTTCTACAGATTGACAAAATGTCGTTGGTGGCCC
	hop 23	hop 23 GGGATCCATGGGCACTATTTATATCAACCCTGATTTTTTCTACAGATTGACAAAATGTCGTTGGTGGCCC
	hop 25	hop 25 GGGATCCATGGGCACTATTTATATCAACCCTGATTTTTTCTACAGATTGACAAAATGTCGTTGGTGGCCC
10 hop	hop 27	hop 27 GGGATCCATGGGCACTATTTATATCAACCCTGATTTTTTCTACAGATTGACAAAATGTCGTTGGTGGCCC
8 apt	hop 32	hop 32 GGGATCCATGGGCACTATTTATATCAACCCTGATTTTTTCTACAGATTGACAAAATGTCGTTGGTGGCCC
	hop 35	hop 35 GGGATCCATGGGCACTATTTATATCAACCCTGATTTTTTCTACAGATTGACAAAATGTCGTTGGTGGCCC
	apt 5	apt5GGGATCCATGGGCACTATTTATATCAACCCTGATTTTTTCTACAGATTGACAAAATGTCGTTGGTGGCCC
	apt 7	apt7GGGATCCATGGGCACTATTTATATCAACCCTGATTTTTTCTACAGATTGACAAAATGTCGTTGGTGGCCC
	apt 15	apt15GGGATCCATGGGCACTATTTATATCAACCCTGATTTTTTCTACAGATTGACAAAATGTCGTTGGTGGCCC
	apt 16	apt16GGGATCCATGGGCACTATTTATATCAACCCTGATTTTTTCTACAGATTGACAAAATGTCGTTGGTGGCCC
	apt 23	apt23GGGATCCATGGGCACTATTTATATCAACCCTGATTTTTTCTACAGATTGACAAAATGTCGTTGGTGGCCC
	apt 26	apt26GGGATCCATGGGCACTATTTATATCAACCCTGATTTTTTCTACAGATTGACAAAATGTCGTTGGTGGCCC
	apt 33	apt33GGGATCCATGGGCACTATTTATATCAACCCTGATTTTTTCTACAGATTGACAAAATGTCGTTGGTGGCCC
	apt 40	apt40GGGATCCATGGGCACTATTTATATCAACCCTGATTTTTTCTACAGATTGACAAAATGTCGTTGGTGGCCC
1 hop	hop 22	hop 22 GGGATCCATGGGCACTATTTATATCAACGCTGATTTTTTCTACAGATTGACAAAATGTCGTTGGTGGCCC
1 hop	hop 42	hop 42 GGGATCCATGGGCACTATTTATATCAACCCTGATTTTTTCTACA-ATTGACAAAATGTCGTTGGTGGCCC
	hop 4	....|....|....|....|....|....|....|....|....|....|....|....|....|....|
	hop 12	hop 4 GGGATCCATGGGCACTATTTATATCAACGGCACACCCTTTAGAAGCAATGCGTAATGTCGTTGGTGGCCC
	hop 13	hop 12 GGGATCCATGGGCACTATTTATATCAACGGCACACCCTTTAGAAGCAATGCGTAATGTCGTTGGTGGCCC
	hop 14	hop 13 GGGATCCATGGGCACTATTTATATCAACGGCACACCCTTTAGAAGCAATGCGTAATGTCGTTGGTGGCCC
	hop 18	hop 14 GGGATCCATGGGCACTATTTATATCAACGGCACACCCTTTAGAAGCAATGCGTAATGTCGTTGGTGGCCC
	hop 28	hop 18 GGGATCCATGGGCACTATTTATATCAACGGCACACCCTTTAGAAGCAATGCGTAATGTCGTTGGTGGCCC
	hop 30	hop 28 GGGATCCATGGGCACTATTTATATCAACGGCACACCCTTTAGAAGCAATGCGTAATGTCGTTGGTGGCCC
11 hop	hop 34	hop 30 GGGATCCATGGGCACTATTTATATCAACGGCACACCCTTTAGAAGCAATGCGTAATGTCGTTGGTGGCCC
9 apt	hop 38	hop 34 GGGATCCATGGGCACTATTTATATCAACGGCACACCCTTTAGAAGCAATGCGTAATGTCGTTGGTGGCCC
	hop 40	hop 38 GAGATCCATGGGCACTATTTATATCAAC GGCACACCCTTTAGAAGCAATGCGTAATGTCGTTGGTGGCCC
	hop 41	hop 40 GGGATCCATGGGCACTATTTATATCAACGGCACACCCTTTAGAAGCAATGCGTAATGTCGTTGGTGGCCC
	apt 4	hop 41 GGGATCCATGGGCACTATTTATATCAACGGCACACCCTTTAGAAGCAATGCGTAATGTCGTTGGTGGCCC
	apt 10	apt 4 GGGATCCATGGGCACTATTTATATCAACGGCACACCCTTTAGAAGCAATGCGTAATGTCGTTGGTGGCCC
	apt 12	apt10GGGATCCATGGGCACTATTTATATCAACGGCACACCCTTTAGAAGCAATGCGTAATGTCGTTGGTGGCCC
	apt 17	apt12GGGATCCATGGGCACTATTTATATCAACGGCACACCCTTTAGAAGCAATGCGTAATGTCGTTGGTGGCCC

	apt 18<br>apt 29<br>apt 38<br>apt 41<br>apt 43	apt17GGGATCCATGGGCACTATTTATATCAACGGCACACCCTTTAGAAGCAATGCGTAATGTCGTTGGTGGCCC<br>apt18GGGATCCATGGGCACTATTTATATCAACGGCACACCCTTTAGAAGCAATGCGTAATGTCGTTGGTGGCCC<br>apt29GGGATCCATGGGCACTATTTATATCAACGGCACACCCTTTAGAAGCAATGCGTAATGTCGTTGGTGGCCC<br>apt38GGGATCCATGGGCACTATTTATATCAACGGCACACCCTTTAGAAGCAATGCGTAATGTCGTTGGTGGCCC<br>apt41GGGATCCATGGGCACTATTTATATCAACGGCACACCCTTTAGAAGCAATGCGTAATGTCGTTGGTGGCCC<br>apt43GGGATCCATGGGCACTATTTATATCAACGGCACACCCTTTAGAAGCAATGCGTAATGTCGTTGGTGGCCC
1 apt	apt 31	apt31GGGATCCATGGGCACTATTTATATCAACAGGCACACCCTTTAGAAGCAATGCGTAATGTCGTTGGTGGCCC
1 hop<br>1 apt	hop 5<br>apt 39	....|....|....|....|....|....|....|....|....|....|....|....|....|....| hop 5 GGGATCCATGGGCACTATTTATATCAACCGATTCTACTTTTCTCAAGGCTCTGAATGTCGTTGGTGGCCC<br>apt39GGGATCCATGGGCACTATTTATATCAACCGATTCTACTTTTCTCAAGGCTCTGAATGTCGTTGGTGGCCC
2 apt	apt 12<br>apt 42	....|....|....|....|....|....|....|....|....|....|....|....|....|....| apt 12GGGATCCATGGGCACTATTTATATCAACGTCACCTGGCCTCTTCGGTCTTCTAAATGTCGTTGGTGGCCC<br>apt42GGGATCCATGGGCACTATTTATATCAACGTCACCTGGCCTCTTCGGTCTTCTAAATGTCGTTGGTGGCCC
1 hop	hop 8	....|....|....|....|....|....|....|....|....|....|....|....|....|....| hop 8 GGGATCCATGGGCACTATTTATATCAACTACTTTGAACGCTTTTTTCTTTTCCAATGTCGTTGGTGGCCC
1 apt	apt 3	....|....|....|....|....|....|....|....|....|....|....|....|....|....| apt 3 GGGATCCATGGGCACTATTTATATCAACATAGCCCTGATTTTTTCTACAGACCAATGTCGTTGGTGGCCC
1 apt	apt 14	....|....|....|....|....|....|....|....|....|....|....|....|....|....| apt 14GGGATCCATGGGCACTATTTATATCAACCGCGCTTTTTTCAGAAGATTGGCGTAATGTCGTTGGTGGCCC
1 apt	apt 27	....|....|....|....|....|....|....|....|....|....|....|....|....|....| apt 27GGGATCCATGGGCACTATTTATATCAACGTACATGCTGCCTATTGGTCTTGTCAATGTCGTTGGTGGCCC
1 apt	apt 32	....|....|....|....|....|....|....|....|....|....|....|....|....|....| apt 32GGGATCCATGGGCACTATTTATATCAACAGCACACCCTATTCAAGCAATGTAAAATGTCGTTGGTGGCCC
1 apt	apt 34	....|....|....|....|....|....|....|....|....|....|....|....|....|....| apt 34GGGATCCATGGGCACTATTTATATCAACTGATTTTTTCTACATTGATATAGCTAATGTCGTTGGTGGCCC
1 apt	apt 37	....|....|....|....|....|....|....|....|....|....|....|....|....|....| apt 37GGGATCCATGGGCACTATTTATATCAACTTCCTGATATTCCTACACTTTTGCAAATGTCGTTGGTGGCCC

Suppl. Table S2. List of primers used for defense related genes RT-qPCR (5'-3'):

AtActin2f: GAAACCCTCGTAGATTGGCA

AtActin2r: CTCTCCCGCTATGTATGTCGC

MEKK1f: TATATGGAGCCTCGGGTGCA

MEKK1r: CAGCAGTTCAGCTGCAGTT

EDS1f: CTGGTACAGTCGATGGGAAAG

EDS1r: TCTTCTATCCATGCTAGTTTC

PAD4f: TTGCCAGTCACCGGAGATGT

PAD4r: GAGATAGAAGCCAAAGTGCGG

PR1f: TCTAAGGGTTCACAACCAGG

PR1r: CCTTCTCGCTAACCCACATG

WRKY40f: CTAGAGACAATCCATCTCCA

WRKY40r: TGCTGCAACGGGTGTTGAAG

Supplementary Figure S1

Binding of aptamers H1 and H2 to the BSA protein (Promega, R3961).The concentration of the fluorescently labeled RNA aptamers is 50nM, the concentration of the BSA protein 0.1 - 10000 nM.

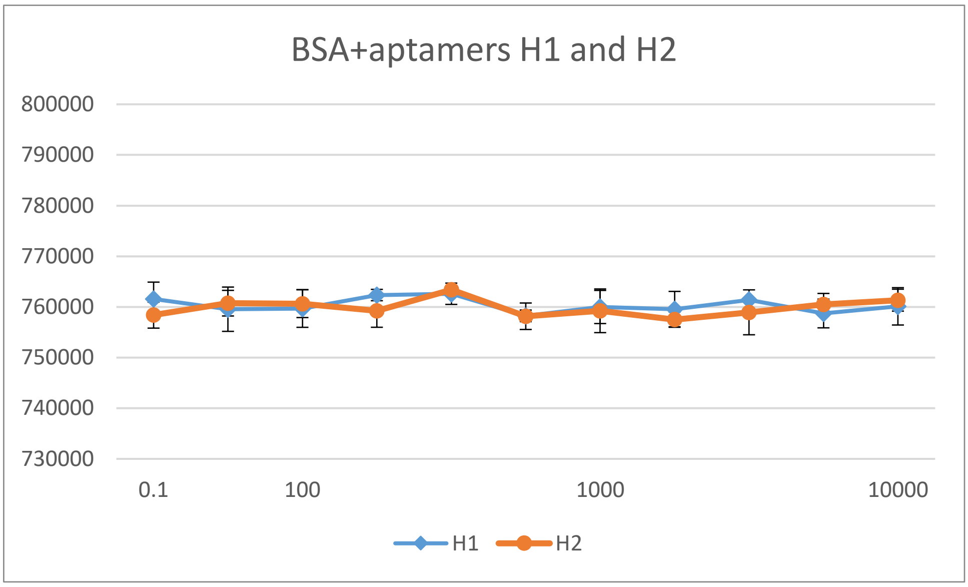

Supplementary Figure S2

Binding of GFP-aptamer and HopU1 protein. The concentration of the fluorescently labeled RNA GFP-aptamer is 50nM, the concentration of the HopU1 protein 0.1 - 10000nM.

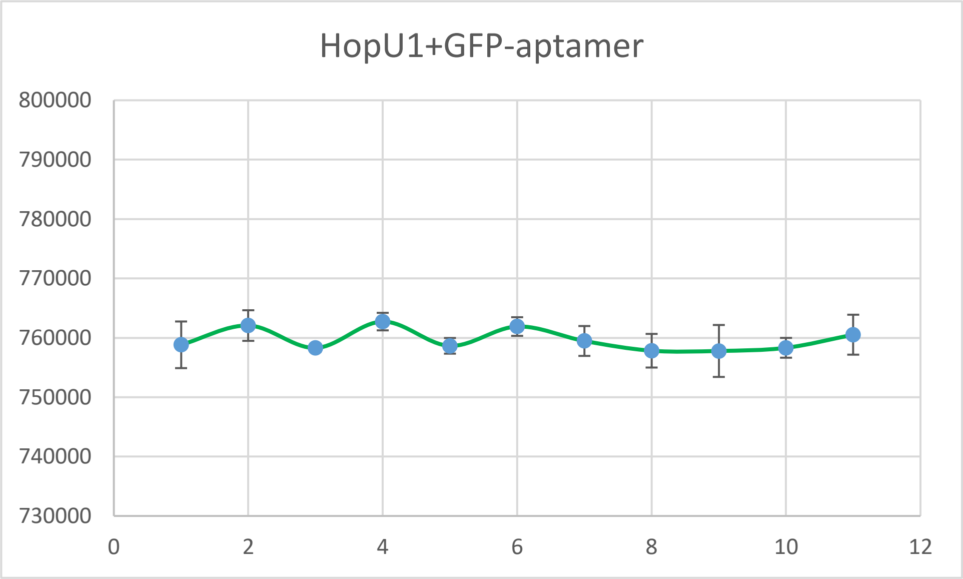

Supplement: Supplementary file 1 [file ijms-24-16604-s001.zip › ijms-2682455-supplementary.pdf]
